# Effect of bariatric surgery on maternal cardiovascular system

**DOI:** 10.1002/uog.26042

**Published:** 2023-02-01

**Authors:** D. Patel, N. Borrelli, O. Patey, M. Johnson, G. DI Salvo, M. D. Savvidou

**Affiliations:** ^1^ Academic Department of Obstetrics and Gynaecology, Chelsea and Westminster Hospital, Department of Metabolism Digestion and Reproduction, Imperial College London London UK; ^2^ Royal Brompton Hospital, Royal Brompton and Harefield NHS Foundation Trust London UK; ^3^ Fetal Medicine Unit, Chelsea and Westminster Hospital London UK

**Keywords:** bariatric surgery, gastric band, gastric bypass, maternal cardiovascular function, obesity, sleeve gastrectomy, weight‐loss surgery

## Abstract

**Objective:**

Bariatric surgery is a successful treatment for sustainable weight loss and has been associated with improvement in cardiovascular function. Pregnancy after bariatric surgery is becoming increasingly common; however, little is known about the maternal cardiovascular system postsurgery. The aim of this study was to investigate maternal cardiovascular adaptation to pregnancy in women with previous bariatric surgery, compared with that in women with no history of weight‐loss surgery and an early‐pregnancy body mass index (BMI) similar to the presurgery BMI of the postbariatric women.

**Methods:**

This was a prospective, observational, longitudinal study conducted from April 2018 to June 2020 including 30 pregnant women who had undergone bariatric surgery and 30 who had not, matched for presurgery BMI. Participants were seen at three timepoints during pregnancy: 12–14, 20–24 and 30–32 weeks' gestation. At all visits, maternal blood pressure (BP) was measured and cardiac geometry and function were assessed using two‐dimensional (2D) transthoracic echocardiography. On a subset of patients (15 in each group), 2D speckle tracking was performed to assess global longitudinal and circumferential strain. Offline analysis was performed, and multilevel linear mixed‐effects models were used for all comparisons.

**Results:**

Compared with the no‐surgery group, and across all trimesters, pregnant women with previous bariatric surgery had lower BP, heart rate and cardiac output and higher peripheral vascular resistance (*P* < 0.01 for all). Similarly, the postbariatric group demonstrated more favorable cardiac geometry and diastolic indices, including lower left ventricular mass, left atrial volume and relative wall thickness, together with higher E‐wave/A‐wave flow velocity across the mitral valve and higher mitral velocity (E′) at the lateral and medial annulus on tissue Doppler imaging (*P* < 0.01 for all). There was no difference in ejection fraction, although global longitudinal strain was lower in postbariatric women (*P* < 0.01), indicating better systolic function.

**Conclusion:**

Our findings indicate better maternal cardiovascular adaptation in women with previous bariatric surgery compared with presurgery BMI‐matched pregnant women with no history of weight‐loss surgery. © 2022 The Authors. *Ultrasound in Obstetrics & Gynecology* published by John Wiley & Sons Ltd on behalf of International Society of Ultrasound in Obstetrics and Gynecology.

**CONTRIBUTION**

*What are the novel findings of this work?*

We found that pregnant women who have undergone bariatric surgery have a better cardiovascular profile than do pregnant women with no history of weight‐loss surgery and an early‐pregnancy body mass index (BMI) similar to the presurgery BMI of the postbariatric women. This is evidenced by more favorable hemodynamic indices, cardiac geometry and diastolic and systolic function.

*What are the clinical implications of this work?*

This study demonstrates that past bariatric surgery is associated with a better maternal cardiovascular profile, which may explain the reduced rate of hypertensive disorders in these pregnancies. These findings may be of importance for obese women of childbearing age considering bariatric surgery.

## INTRODUCTION

Obesity rates are growing worldwide and, in the UK, a third of women were classified as obese in 2018^1^. Obesity is associated with an increase in circulating blood volume, left ventricular hypertrophy and impaired diastolic and systolic function^2^. Bariatric surgery is the most effective method for inducing long‐lasting weight loss^3^ and is currently recommended for individuals with a body mass index (BMI) ≥ 40 kg/m^2^ or for those with a BMI of 35–39.9 kg/m^2^ presenting with comorbidities such as diabetes or hypertension^4^, ^5^. Studies investigating the effect of bariatric surgery on the cardiovascular system outside pregnancy have found that surgery is associated with an improvement or resolution in hypertension, changes in cardiac geometry and an improvement in diastolic and systolic function^6^, ^7^, ^8^.

Normal pregnancy is accompanied by several physiological changes to the maternal cardiovascular system, including an increase in stroke volume (SV) and cardiac output (CO) and a reduction in peripheral vascular resistance (PVR)^9^. Typically, maternal ejection fraction remains unchanged but there is a tendency towards reduced diastolic reserve with physiological left ventricular hypertrophy^10^. Maternal cardiovascular function has been implicated in the pathophysiology of placenta‐related complications, such as pre‐eclampsia (PE) and fetal growth restriction^11^, ^12^, ^13^, and several studies have shown that women destined to develop late‐onset PE have a high CO, left ventricular hypertrophy and reduced diastolic indices^12^, ^14^, ^15^, changes also seen in obese pregnant women^15^, ^16^, ^17^, ^18^.

Owing to the increasing prevalence of obesity, the number of women entering pregnancy having undergone bariatric surgery is also increasing. There is now good evidence that, compared with obese pregnant women managed conservatively, pregnancy following bariatric surgery is associated with a reduced risk of hypertensive disorders^19^, which are known to be closely linked to maternal cardiac performance. Limited research exists on the maternal cardiovascular adaption of pregnant women with previous bariatric surgery. We reported previously that postbariatric pregnant women have a favorable cardiovascular profile compared with pregnant women who have not undergone surgery, matched for early‐pregnancy BMI^20^. Matching for early‐pregnancy BMI assesses the effect of surgery once the weight has been lost, whereas matching for presurgery BMI informs of the effect of weight loss and surgery. In the current study, we aimed to investigate the cardiovascular profile of pregnant women with previous bariatric surgery, compared with that of women with no history of weight‐loss surgery and early‐pregnancy BMI similar to the presurgery BMI of their postbariatric counterparts.

## METHODS

This was a prospective, observational, longitudinal study conducted from April 2018 to June 2020. Women with a singleton pregnancy were identified through a perinatal database, approached soon after their first‐trimester scan and recruited to the following groups: (1) pregnant women with previous bariatric surgery or (2) pregnant women with no history of bariatric surgery, matched for presurgery BMI, age, race and diabetes status. All participants were seen at three timepoints during their pregnancy: 12–14, 20–24 and 30–32 weeks' gestation.

The study protocol has been described previously^20^. In brief, at all visits maternal height and weight were measured to the nearest 0.5 cm and 0.1 kg, respectively, and BMI was calculated in kg/m^2^. Gestational diabetes mellitus (GDM) testing was undertaken at 28–30 weeks' gestation; women with previous bariatric surgery underwent home glucose monitoring for 2 weeks and were diagnosed if they had persistently raised fasting (≥ 5.3 mmol/L) and/or postmeal (≥ 7.8 mmol/L) capillary glucose levels^21^. Those with no previous bariatric surgery underwent a 2‐h 75‐g oral glucose tolerance test, and diabetes was diagnosed if the fasting plasma glucose level was ≥ 5.6 mmol/L and/or the 2‐h plasma glucose level was ≥ 7.8 mmol/L^21^. Delivery outcomes and birth weight were obtained from the hospital database and birth‐weight percentiles were calculated^22^.

Maternal blood pressure (BP) measurements were obtained manually using a sphygmomanometer (Accoson Dekamet; AC Cossor & Son (Surgical) Ltd, London, UK). Two readings from the left arm were taken 5 min apart and the mean value was recorded. Mean arterial pressure (MAP) (mmHg) was calculated as (systolic BP + (2 ×diastolic BP))/3^23^. Transthoracic echocardiography was used to assess the maternal cardiovascular system with two‐dimensional, M‐mode and tissue Doppler imaging (TDI) using an iE33 system (Philips Ultrasound, Bothell, WA, USA) according to European and American guidelines^24^, ^25^. Standard parasternal and apical views were used and digital loops of three cardiac cycles with associated electrocardiographic information were obtained. CO (L/min) was calculated as SV × heart rate (HR) (bpm)^26^. SV (mL) was calculated as the cross‐sectional area of the left ventricular outflow tract × the velocity time integral^26^. PVR (dynes × s/cm^5^) was calculated as MAP × 80/CO^17^, ^23^. Left ventricular mass (g) was calculated as 0.8 × (1.04 × ((interventricular septum diameter (mm) + left ventricle internal diameter (mm) +posterior wall thickness (mm))^3^ – left ventricle internal diameter^3^ (mm))) + 0.6. Relative wall thickness (RWT) was calculated as (2 × posterior wall thickness (mm)) / left ventricle internal diameter (mm)^24^. All measurements were taken in diastole. Body surface area (m^2^) was calculated^27^ as (weight (kg))^0.425^ × (height (cm))^0.725^ ×0.007184. Global longitudinal strain (GLS) and global circumferential strain (GCS) were calculated in a subgroup of patients (15 in each group) using speckle‐tracking analysis. Parasternal short‐axis views and apical two‐, three‐ and four‐chamber views were analyzed using 2D Cardiac Performance Analysis (TomTec Imaging System, Munich, Germany).

Hemodynamic function was assessed by systolic BP (SBP), diastolic BP (DBP), MAP, HR, SV, CO and PVR. Cardiac geometry was evaluated by left atrial diameter (end‐systole), interventricular septum thickness (end‐diastole), left ventricle diameter (end‐diastole), posterior wall thickness (end‐diastole), RWT and left ventricular mass. Diastolic function was assessed by the ratio of the mitral peak early (E) and late (A) diastolic flow velocities (E/A ratio), lateral (E′ lat) and medial (E′ med) mitral annular velocities measured on TDI, E/E′ ratio (where E′ is the mean of E′ lat and E′ med) and left atrial volume. Systolic function was assessed by end‐diastolic volume, end‐systolic volume, ejection fraction and peak systolic velocity at the lateral tricuspid annulus measured on TDI (S′). GLS and GCS provided further systolic function assessment. All echocardiographic data were stored for offline analysis.

Echocardiographic studies and speckle‐tracking analyses were performed by experienced operators (D.P., N.B. and O.P.), who were blinded to the allocation of study participants. The study was approved by the local research ethics committee (reference number 14/LO/0592) and all women provided written consent.

### Statistical analysis

Normality of the data was assessed by the Kolmogorov–Smirnov test. Data are expressed as mean ± SD for normally distributed data, median (interquartile range) for non‐normally distributed data and as *n* (%) for categorical variables. Numerical and categorical data were compared using the unpaired *t*‐test/Mann–Whitney *U*‐test and χ‐square test, respectively.

Log_10_ transformation was performed for non‐parametric data and multilevel linear mixed‐effects models were used to compare the groups. The fixed‐effect component included timepoint (three study visits), study group, age, race, smoking, gestational age, development of GDM and first‐order interaction between time and study group. Statistical analysis was performed using Statistical Package for the Social Sciences (SPSS) for Windows 2019, version 26.0 (IBM Corp., Armonk, NY, USA); *P* < 0.05 was considered to indicate statistical significance.

Sample size calculation was performed using G*Power software (G*Power for Windows OS X, version 3.1; Heinrich‐Heine‐Universität, Dusseldorf, Germany)^28^. There are no prior studies investigating the cardiovascular system of pregnant women with previous bariatric surgery compared with women with similar presurgery BMI. Therefore it was difficult to estimate accurately the number of subjects required in each group to obtain results with adequate power. Using studies of individuals before and after bariatric surgery, outside the context of pregnancy, we estimated sample sizes for an alpha‐level of 0.05 and a power of 80%. In order to detect a mean difference of 1.4 mL/min in CO^29^, 0.32 in E/A ratio and 5.6% in GLS, sample sizes of 23, 22 and five women, respectively, would have been needed in each group^30^.

## RESULTS

**Figure 1:**
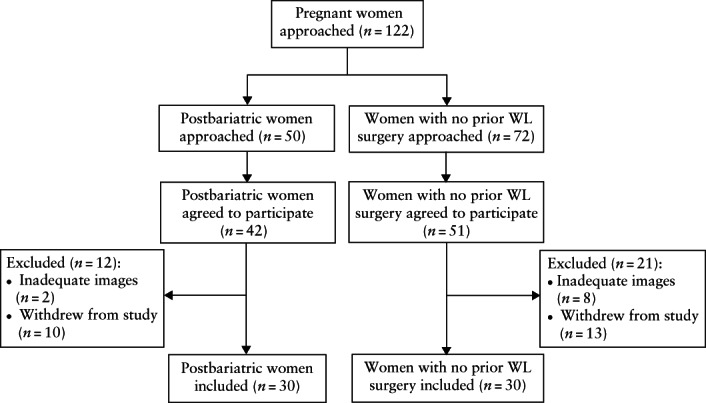
Flowchart summarizing patient inclusion in study. WL, weight loss.

Of 122 pregnant women (50 postbariatric and 72 with no previous weight‐loss surgery) approached, 42 postbariatric women and 51 women with no previous weight‐loss surgery agreed to participate (Figure 1). Those who attended at least two of the three research visits, with known pregnancy outcome, were included, and the resulting cohort had 30 women in each group, matched closely for presurgery BMI, age and race. The maternal characteristics of the study participants are given in Table 1. In the postbariatric group, one woman had undergone gastric‐band surgery, 14 had undergone sleeve gastrectomy and 15 had undergone gastric bypass. The mean surgery‐to‐conception interval was 48.8 ± 32.3 months and the mean weight loss from presurgery to early pregnancy was 29.5 ± 17.5 kg.

**Table 1:** Maternal demographic characteristics and pregnancy outcomes of 30 women who had undergone bariatric surgery and 30 women with no history of weight‐loss surgery

Variable	No surgery (*n* = 30)	Bariatric surgery (*n* = 30)	*P*
Age (years)	32.5 ± 5.2	33.4 ± 6.1	0.88
Race			0.74
White	24 (80.0)	25 (83.3)	
Other	6 (20.0)	5 (16.7)	
BMI at presentation (kg/m^2^)	42.4 ± 5.9	37.3 ± 7.8	< 0.01
Presurgery BMI (kg/m^2^)	—	43.0 ± 5.9	0.66*
Parity			0.02
Nulliparous	11 (36.7)	20 (66.7)	
Parous	19 (63.3)	10 (33.3)	
Conception			1.00
Spontaneous	29 (96.7)	29 (96.7)	
Assisted	1 (3.3)	1 (3.3)	
Smoker	2 (6.7)	3 (10.0)	0.64
Hypertensive disorder of pregnancy	3 (10.0)	0 (0)	< 0.001
Gestational diabetes mellitus	7 (23.3)	5 (16.7)	0.52
Mode of delivery			
Vaginal delivery	15 (50.0)	17 (56.7)	0.69
Cesarean section	15 (50.0)	13 (43.3)	0.16
GA at delivery (weeks)	39.1 (38.1–40.1)	39 (37.5–39.7)	0.54
Birth weight (g)	3550 (3065–3832)	3160 (2490–3357)	0.01
Birth‐weight percentile	75 (35.7–91.7)	30 (9.6–49.7)	< 0.01

Data are expressed as mean ± SD, *n* (%) or median (interquartile range).

* Presurgery body mass index (BMI) in postbariatric group *vs* early‐pregnancy BMI in no‐surgery group.

GA, gestational age.

There were no significant differences in maternal demographic characteristics between study groups, with the exception of BMI at presentation (Table 1), as expected. None of the women in the postbariatric group developed hypertensive disorders and, in keeping with the literature, postbariatric women delivered smaller babies^19^.

### Hemodynamic parameters

**Figure 2:**
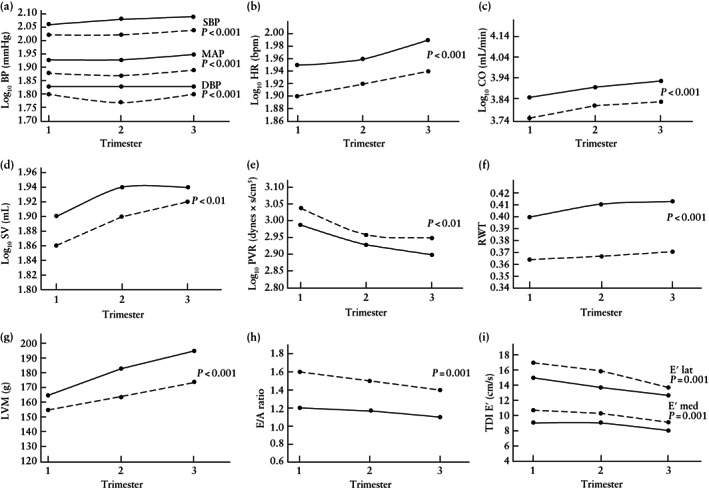
Mixed‐effects model analysis across all trimesters in 30 pregnant women who had undergone bariatric surgery (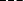) and 30 women with no history of weight‐loss surgery (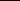) for: (a) log_10_ blood pressure (BP), (b) log_10_ heart rate (HR), (c) log_10_ cardiac output (CO), (d) log_10_ stroke volume (SV), (e) log_10_ peripheral vascular resistance (PVR), (f) relative wall thickness (RWT), (g) left ventricular mass (LVM), (h) E/A ratio and (i) E′ at lateral (E′ lat) and medial (E′ med) mitral annulus on tissue Doppler imaging (TDI). DBP, diastolic blood pressure; MAP, mean arterial pressure; SBP, systolic blood pressure.

Mixed‐effects model analysis was used to compare the log_10_‐transformed hemodynamic parameters between the postbariatric and no‐surgery groups overall (Table 2) and at each trimester (Tables S1 and S2). Women with previous bariatric surgery had lower SBP, DBP and MAP throughout gestation than did the no‐surgery group (Figure 2a). Similarly, postbariatric women had lower HR and SV, which resulted in lower CO, with higher PVR (Figure 2b–e). In the whole cohort, maternal BMI was correlated positively with SBP and CO across all three trimesters (Table 3). Similarly, in the whole cohort as well as in the no‐surgery group, maternal CO and PVR were correlated positively and negatively, respectively, with birth‐weight percentile, but this was not the case in postbariatric women (data not shown).

**Table 2:** Multilevel linear mixed‐effects models comparing log_10_‐transformed hemodynamic parameters, cardiac geometry and systolic and diastolic function, across all trimesters, between 30 pregnant women who had undergone bariatric surgery and 30 with no history of weight‐loss surgery

Parameter	Mean difference (95% CI)*	*P*
Hemodynamic parameter		
Log_10_ SBP (mmHg)	0.047 (0.033 to 0.061)	< 0.001
Log_10_ DBP (mmHg)	0.04 (0.024 to 0.057)	< 0.001
Log_10_ MAP (mmHg)	0.043 (0.029 to 0.057)	< 0.001
Log_10_ HR (bpm)	0.05 (0.032 to 0.068)	< 0.001
Log_10_ SV (mL)	0.042 (0.015 to 0.068)	< 0.01
Log_10_ CO (L/min)	0.093 (0.065 to 0.12)	< 0.001
Log_10_ PVR (dynes×s/cm^5^)	–0.051 (–0.079 to –0.022)	< 0.01
Log_10_ SV index (mm/m^2^)†	0.009 (–0.016 to 0.035)	0.46
Log_10_ cardiac index (mm/m^2^)†	0.061 (0.036 to 0.085)	< 0.001
Cardiac geometry		
LA (mm)	0.606 (–1.368 to 2.581)	0.55
LA index (mm/m^2^)†	0.837 (–0.072 to 1.603)	0.08
IVS (mm)	0.7 (0.304 to 1.096)	< 0.01
LVEDD (mm)	0.291 (–1.792 to 1.209)	0.70
LVEDD index (mm/m^2^)†	1.476 (–0.335 to 2.617)	0.05
PW (mm)	0.939 (0.545 to 1.333)	< 0.001
RWT	0.044 (0.023 to 0.065)	< 0.001
LVM (g)	16.976 (6.22 to 27.732)	< 0.01
LVM index (g/m^2^)†	3.581 (–1.214 to 8.375)	0.14
Systolic function		
EDV (mm)	4.706 (–0.922 to 10.334)	0.01
EDV index (mm/m^2^)†	–1.561 (–4.021 to 0.898)	0.21
ESV (mm)	3.062 (0.272 to 5.851)	0.03
ESV index (mm/m^2^)†	–0.068 (–1.298 to 1.161)	0.91
EF (%)	–0.668 (–2.167 to 0.831)	0.38
S′ (cm)	0.056 (–0.798 to 0.909)	0.90
Diastolic function		
E/A ratio	–0.271 (–0.37 to –0.172)	< 0.001
E′ lat (cm/s)	–1.806 (–2.773 to –0.838)	< 0.001
E′ med (cm/s)	–1.303 (–2.024 to –0.583)	< 0.001
E/E′ ratio	0.664 (0.099 to 1.23)	0.02
LAV (mL)	6.255 (2.592 to 9.918)	< 0.01
LAV index (mL/m^2^)†	0.578 (–1.122 to 2.277)	0.50
Strain		
GLS (%)	2.20 (0.56 to 3.85)	< 0.01
GCS (%)	1.64 (–0.73 to 4.00)	0.17

* No surgery – past bariatric surgery.

† Indexed to body surface area.

CO, cardiac output; DBP, diastolic blood pressure; E′ lat, peak early diastolic flow velocity measured on tissue Doppler imaging (TDI) at lateral mitral annulus; E′ med, peak early diastolic flow velocity measured on TDI at medial mitral annulus; E/A ratio, ratio of mitral peak early and late diastolic flow velocities; E/E′ ratio, ratio of mitral peak early diastolic flow velocity to mean value of E′ lat and E′ med; EDV, end‐diastolic volume; EF, ejection fraction; ESV, end‐systolic volume; GCS, global circumferential strain; GLS, global longitudinal strain; HR, heart rate; IVS, interventricular septal thickness; LA, left atrial diameter; LAV, left atrial volume; LVEDD, left ventricular end‐diastolic diameter; LVM, left ventricular mass; MAP, mean arterial pressure; PVR, peripheral vascular resistance; PW, posterior wall thickness; RWT, relative wall thickness; SBP, systolic blood pressure; SV, stroke volume; S′, peak systolic velocity at lateral tricuspid annulus on TDI.

**Table 3:** Correlation between body mass index and cardiac parameters in 30 pregnant women who had undergone bariatric surgery and 30 with no history of weight‐loss surgery

	First trimester		Second trimester		Third trimester	
Parameter	*r*	*P*	*r*	*P*	*r*	*P*
SBP (mmHg)	0.46	< 0.01	0.25	< 0.05	0.40	< 0.01
DBP (mmHg)	0.24	0.10	0.10	0.46	0.33	0.01
HR (bpm)	0.17	0.25	0.26	0.05	0.20	0.13
SV (mL)	0.20	0.18	0.16	0.21	0.28	0.04
CO (L/min)	0.27	< 0.05	0.35	0.01	0.42	< 0.01
LVM (g)	0.35	0.02	0.20	0.12	0.14	0.32

CO, cardiac output; DBP, diastolic blood pressure; HR, heart rate; LVM, left ventricular mass; *r*, Pearson's correlation coefficient; SBP, systolic blood pressure; SV, stroke volume.

### Cardiac geometry

Postbariatric women had lower interventricular septal thickness, posterior wall diameter, left ventricular mass and RWT than did their no‐surgery counterparts, in all trimesters (Table 2, Table S1 and Figure 2f,g). In the whole cohort, maternal BMI was correlated positively with left ventricular mass (significant only in the first trimester) (Table 3).

### Systolic, diastolic and longitudinal function

In the overall analysis across trimesters, the postbariatric group had lower end‐diastolic and end‐systolic volumes than did the no‐surgery group, but there was no difference in ejection fraction or TDI‐S′ at the lateral tricuspid annulus (Table 2). For diastolic indices, the postbariatric group had higher E/A ratio and TDI‐E′ lat and E′ med, and lower E/E′ ratio and left atrial volume, suggesting better diastolic function (Table 2 and Figure 2h,i).

### Global strain

GLS was lower in the postbariatric group than in the no‐surgery group, suggesting better systolic function in the former (Table 2).

All of the above results were unchanged when women with hypertensive disorders (*n* = 3, all in the no‐surgery group) were excluded from the analyses.

## DISCUSSION

We found that pregnant women with previous bariatric surgery had improved hemodynamic profile, cardiac geometry and systolic and diastolic indices, compared with pregnant women who had not undergone surgery and whose early‐pregnancy BMI was similar to the presurgery BMI of the postbariatric women. In detail, postbariatric women demonstrated lower BP, HR, SV and CO, higher PVR and lower left ventricular mass and RWT. Similarly, diastolic indices were more favorable, including higher E/A ratio and TDI‐E′ lat and E′ med, and lower E/E′ ratio and left atrial volume. There was no difference in ejection fraction, but GLS was lower in the postbariatric group, indicating improved systolic function.

We reported recently that women with previous bariatric surgery have improved cardiovascular adaptation to pregnancy compared with women with no history of bariatric surgery, matched for early‐pregnancy BMI^20^. We have now extended our work by matching for presurgery BMI. We found that in the postbariatric group, maternal BP was lower. Obesity is associated with increased plasma volume expansion and CO owing to excess body mass, with a concomitant decrease in natriuresis^31^. More recently, it has been suggested that neurohormonal factors play a role in the control of BP and several studies have shown a reduction or resolution in hypertension after bariatric surgery^6^. Evidence suggests that higher maternal BP in the first trimester of pregnancy is associated with a higher risk of hypertensive disorders later on^32^, therefore the lower BP found in postbariatric women may provide a plausible explanation for the reduced rates of hypertension and PE reported in this population^19^, ^33^. In this study, CO increased with gestation in both groups, reflecting physiological pregnancy changes^10^; however, in the postbariatric pregnant group, lower SV and HR resulted in lower CO, as observed in postbariatric individuals outside the context of pregnancy^29^, ^34^.

With regard to cardiac geometry, the postbariatric group demonstrated lower left ventricular mass and RWT than did the no‐surgery group, consistent with findings in non‐pregnant individuals after bariatric surgery^7^, ^29^, ^35^. In addition, maternal BMI was correlated positively with left ventricular mass. The traditional hemodynamic model in obesity describes increased stroke workload leading to left ventricular dilatation and increasing myocardial mass to compensate, with subsequent left ventricular diastolic and, sometimes, systolic, dysfunction. More recent thinking is that early hypertrophic heart changes are secondary to obesity‐associated hyperleptinemia, and that the subsequent cardiac dilatation seen in morbid obesity is likely to be volume‐induced^36^. In our study, the no‐surgery group showed a tendency towards concentric left ventricle remodeling (RWT, 0.40), which was not the case for the postbariatric group (RWT, 0.36). Of note, the measures of cardiac geometry, including RWT and left ventricular mass, seen in our postbariatric population were similar to those described in normal‐weight pregnant women with a BMI at presentation of 23 kg/m^2^ (RWT, 0.33–0.36)^10^. Considering that our postbariatric group had a mean BMI of 37 kg/m^2^, this finding strongly supports the notion that the beneficial effects of surgery may extend beyond the impact of weight loss alone.

There was no difference in ejection fraction between the groups, however GLS was lower in postbariatric women, suggesting better systolic function. Diastolic indices were also more favorable in the postbariatric group, with higher E/A ratio and TDI‐E′ lat and E′ med, and lower E/E′ ratio and left atrial volume, which is consistent with findings in non‐pregnant individuals following bariatric surgery^7^, ^30^, ^37^, ^38^.

The improvements seen in many cardiovascular indices in the postbariatric women are likely to be due not only to weight loss but also to surgery‐induced gut hormone manipulation, including improved insulin resistance^39^, reduction in leptin and aldosterone production^40^ and increased glucagon‐like peptide‐1 (GLP‐1) levels^41^. Amelioration of insulin sensitivity can decrease renal sodium reabsorption and sympathetic nervous system (SNS) activity^42^, ^43^, with improvement in cardiac and sympathetic baroreflex function^44^, leading to lower maternal BP, HR, SV and, eventually, CO. Decreased leptin levels, which can downregulate SNS activity^45^ and reduce left ventricular hypertrophy, have also been implicated in the reduction of BP and left ventricular mass and beneficial changes in diastolic cardiac indices, as seen following bariatric surgery^6^, ^36^, ^46^. Similarly, reduction in the level of aldosterone, which is known to modulate vascular tone and reduce compliance through the promotion of vascular collagen deposition and remodeling, is likely to play a role in the lower BP seen after surgery^47^, ^48^. Finally, increased levels of GLP‐1, which has cardioprotective properties against endothelial dysfunction, anti‐inflammatory effects on macrophages and antiproliferative effects on smooth muscle cells, may have contributed to the better diastolic indices seen in the postbariatric group^7^, ^36^, ^49^, ^50^, ^51^, ^52^.

The prevalence of PE has been shown to be lower in women with previous bariatric surgery^19^, and studies of PE have shown altered hemodynamic function and cardiac geometry as well as reduced diastolic function prior to the clinical phase of the condition^12^, ^14^, ^15^. The improved cardiac profile of postbariatric pregnant women noted in this study may contribute to the reduced prevalence of hypertensive disorders in this population.

A strength of this study is that it provides novel data on the cardiovascular system in pregnant women with previous bariatric surgery compared with women with similar presurgery BMI, and therefore attempts to simulate the pre‐ and postsurgery states. The longitudinal study design and the use of experienced operators add strength to our findings. Although the number of women who did not undergo surgery is small, the groups were matched closely for presurgery BMI, age, race and diabetes status. We did not have information on the presurgery or prepregnancy cardiac status of our population, and these factors may have affected the cardiovascular profile during pregnancy. Owing to the relatively small number of women in the postbariatric group, we were unable to assess maternal cardiac function according to the type of surgery performed.

In summary, our study found significant differences in the hemodynamic indices, cardiac geometry and systolic and diastolic function of pregnant women with and without a history of bariatric surgery, matched for presurgery BMI. The findings support the beneficial effects of bariatric surgery on maternal cardiovascular health and suggest improved adaptation to pregnancy in postbariatric women. Our results may provide an explanation for the reduced risk of pregnancy‐related hypertension in these women.

## Supporting information


**Table S1** Multilevel linear mixed‐effects models comparing log_10_‐transformed hemodynamic parameters, cardiac geometry and systolic and diastolic function between no‐surgery and postsurgery groups at each trimester
**Table S2** Hemodynamic parameters in no‐surgery and postsurgery groups at each trimester

## Data Availability

The data that support the findings of this study are available from the corresponding author upon reasonable request.
