# Supplementary material for: Effect of bariatric surgery on maternal cardiovascular system
Source: Ultrasound Obstet Gynecol. 2023 Feb 1;61(2):207–14. doi: 10.1002/uog.26042 (PMC10107918; doi:10.1002/uog.26042)
Supplement: Supplementary file 1 — Table S1 Multilevel linear mixed‐effects models comparing log10‐transformed hemodynamic parameters, cardiac geometry and systolic and diastolic function between no‐surgery and postsurgery groups at each trimester Table S2 Hemodynamic parameters in no‐surgery and postsurgery groups at each trimester [file UOG-61-207-s001.docx]

**Table S1** Multilevel linear mixed-effects models comparing log10-transformed hemodynamic parameters, cardiac geometry and systolic and diastolic function between no-surgery and postsurgery groups at each trimester

|  | **1st Trimester** | | | **2nd Trimester** | | | **3rd Trimester** | | |
| --- | --- | --- | --- | --- | --- | --- | --- | --- | --- |
|  | **No-surgery** | **Post-bariatric surgery** | **P** | **No-surgery** | **Post-bariatric surgery** | **P** | **No-surgery** | **Post-bariatric surgery** | **P** |
|  | **N=18** | **N=18** |  | **N=30** | **N=30** |  | **N=28** | **N=28** |  |
| **Haemodynamic** |  |  |  |  |  |  |  |  |  |
| Log10 SBP (mmHg) | 2.06 (2.04 -2.11) | 2.02 (1.99 -2.05) | **<0.001** | 2.08 (2.03 -2.08) | 2.02 (1.99 -2.04) | **<0.01** | 2.09 (2.04 -2.09) | 2.04 (2 -2.05) | **<0.001** |
| Log10 DBP (mmHg) | 1.83 (1.8 -1.87) | 1.8 (1.76 -1.83) | **0.02** | 1.83 (1.8 -1.85) | 1.77 (1.75 -1.8) | **<0.001** | 1.83 (1.81 -1.86) | 1.8 (1.78 -1.83) | **0.04** |
| Log 10 MAP (mmHg) | 1.93 (1.9 -1.96) | 1.88 (1.86 -1.91) | **<0.01** | 1.93 (1.9 -1.94) | 1.87 (1.85 -1.89) | **<0.001** | 1.95 (1.9 -1.97) | 1.89 (1.87 -1.92) | **0.01** |
| Log10 HR (bpm) | 1.95 (1.92 -1.99) | 1.9 (1.86 -1.93) | **<0.01** | 1.96 (1.93 -1.99) | 1.92 (1.88 -1.95) | **<0.01** | 1.99 (1.96 -2.02) | 1.94 (1.9 -1.97) | **<0.01** |
| Log10 SV (ml) | 1.9 (1.85 -1.95) | 1.86 (1.81 -1.91) | **0.04** | 1.94 (1.88 -1.98) | 1.9 (1.85 -1.94) | **0.04** | 1.94 (1.89 -1.98) | 1.92 (1.89 -1.95) | 0.13 |
| Log10 CO (L/min) | 3.85 (3.8 -3.9) | 3.75 (3.7 -3.81) | **<0.001** | 3.9 (3.85 -3.94) | 3.81 (3.76 -3.86) | **<0.01** | 3.93 (3.88 -3.98) | 3.83 (3.78 -3.88) | **<0.001** |
| Log10 PVR (dynes/s per cm^5^) | 2.99 (2.93 -3.04) | 3.04 (2.98 -3.09) | **0.04** | 2.93 (2.88 -2.98) | 2.96 (2.91 -3.01) | 0.15 | 2.9 (2.85 -2.95) | 2.95 (2.91 -3.02) | **0.01** |
| Log10 SVI (mm/BSA) | 1.55 (1.5 -1.6) | 1.53 (1.48 -1.58) | 0.37 | 1.58 (1.54 -1.63) | 1.57 (1.53 -1.62) | 0.69 | 1.58 (1.53 -1.62) | 1.57 (1.53 -1.62) | 0.93 |
| Log10 CI (mm/BSA) | 3.5 (3.45 -3.55) | 3.43 (3.38 -3.48) | **<0.01** | 3.54 (3.5 -3.58) | 3.49 (3.45 -3.53) | **0.02** | 3.57 (3.53 -3.62) | 3.51 (3.46 -3.55) | **0.01** |
| **Cardiac geometry** |  |  |  |  |  |  |  |  |  |
| LA (mm) | 39 (37.2 -42.8) | 37.6 (34.8 -40.4) | 0.12 | 39.5 (37.5 -42.4) | 38.7 (37.2 -42.2) | 0.85 | 40 (37.5 -42.4) | 38.9 (36.1 -41.3) | 0.28 |
| LA index (mm/BSA) | 17.7 (16.3 -19.2) | 17.8 (16.4 -19.3) | 0.84 | 17.6 (16.3 -18.9) | 18 (17.7 -20.4) | 0.09 | 18.9 (16.2 -18.9) | 18.5 (17.1 -19.9) | 0.19 |
| IVS (mm) | 9.7 (8.9 -10.4) | 8.9 (8.2 -9.7) | **<0.05** | 10 (9.4 -10.7) | 9.3 (8.6 -10) | **0.02** | 9.9 (9.3 -10.6) | 9.3 (8.6 -10) | **0.03** |
| LVEDD (mm) | 47.9 (44.1 -49.7) | 48.3 (45.4 -51.2) | 0.31 | 49.2 (46.6 -51.7) | 48.9 (46.3 -51.6) | 0.8 | 50.4 (47.7 -53) | 50.1 (47.3 -52.9) | 0.86 |
| LVEDD index (mm/BSA) | 20.5 (18.2 -22.8) | 21.7 (19.4 -24) | 0.12 | 21.3 (19.8 -22.9) | 23.2 (21.6 -24.8) | **0.06** | 22.3 (20.8 -23.9) | 23.7 (22 -25.3) | 0.08 |
| PW (mm) | 9.8 (9.1 -10.5) | 8.9 (8.2 -9.6) | **<0.05** | 9.9 (9.3 -10.6) | 9 (8.3 -9.7) | **<0.01** | 10.3 (9.7 -10.9) | 9.4 (8.7 -10) | **<0.01** |
| RWT | 0.40 (0.38-0.46) | 0.36 (0.33-0.41) | **0.03** | 0.41 (0.37-0.44) | 0.36 (0.33-0.40) | **0.02** | 0.41 (0.38-0.45) | 0.37 (0.33-0.41) | **0.01** |
| LVM (g) | 165 (145.9 -184.2) | 154.8 (135.1 -174.6) | **0.03** | 183.1 (164.5 -201.7) | 164 (144.6 -183.3) | **0.02** | 195.2 (175.5 -214.9) | 173.6 (152.9 -194.3) | **0.04** |
| LVM index (g/BAS) | 72.5 (64.2 -80.8) | 72.2 (63.6 -80.8) | 0.84 | 80.3 (72.2 -88.4) | 77.3 (68.9 -85.7) | 0.29 | 86.6 (77.6 -95.7) | 79.2 (69.9 -88.5) | 0.11 |
| **Systolic function** | | | | | | | | | |
| EDV (mm) | 95.3 (83.8 -106.8) | 93.1 (81.8 -104.4) | 0.78 | 99.8 (90.1 -109.5) | 95.1 (84.8 -105.4) | 0.32 | 103.8 (93.7 -113.8) | 96.6 (85.8 -107.4) | 0.12 |
| EDV index (mm/BSA) | 41.7 (36.6 -46.7) | 44.4 (39.4 -49.3) | 0.19 | 43.8 (39.6 -48.1) | 45.5 (41 -50) | 0.37 | 45.5 (41.3 -49.7) | 45.8 (41.3 -50.3) | 0.97 |
| ESV (mm) | 37.6 (32.1 -43.2) | 35.4 (30 -40.8) | 0.49 | 40.3 (35.1 -45.5) | 37.2 (31.7 -42.7) | 0.23 | 42.9 (37.7 -48.1) | 39 (33.4 -44.6) | 0.1 |
| ESV index (mm/BSA) | 16.5 (14 -18.9) | 16.9 (14.5 -19.3) | 0.43 | 17.7 (15.4 -20) | 17.8 (15.4 -20.3) | 0.89 | 18.9 (16.7 -21.2) | 18.5 (16.1 -20.9) | 0.57 |
| EF (%) | 60 (57 -63) | 61 (58.1 -64) | 0.72 | 59.3 (56.6 -62) | 60.7 (57.8 -63.6) | 0.23 | 59.9 (57.1 -62.7) | 59.4 (56.4 -62.4) | 0.77 |
| TDI s' (cm) | 13.3 (11.6 -15) | 13.2 (11.5 -14.8) | 0.86 | 13.5 (12.2 -14.8) | 13.9 (12.4 -15.3) | 0.58 | 13.3 (11.9 -14.6) | 12.9 (11.4 -14.3) | 0.6 |
| **Diastolic function** | | | | | | | | | |
| E/A ratio | 1.2 (1 -1.4) | 1.6 (1.3 -1.7) | **<0.01** | 1.2 (1.1 -1.4) | 1.5 (1.3 -1.7) | **<0.001** | 1.1 (0.9 -1.3) | 1.4 (1.2 -1.5) | **<0.001** |
| E' lateral (m/s) | 15 (13 -17) | 17 (15.1 -19) | **0.02** | 13.7 (12 -15.3) | 15.9 (14.2 -17.7) | **0.01** | 12.7 (11 -14.3) | 13.8 (12 -15.5) | 0.16 |
| E' medial (m/s) | 9.1 (7.7 -10.4) | 10.7 (9.3 -12.1) | **0.02** | 9.1 (7.9 -10.3) | 10.3 (9 -11.5) | **0.03** | 8.1 (6.9 -9.4) | 9.2 (7.9 -10.5) | 0.12 |
| E/ E' ratio | 6.9 (6 -7.9) | 6.2 (5.3 -7.2) | 0.16 | 7.6 (6.7 -8.5) | 7 (6 -7.9) | 0.1 | 7.7 (6.7 -8.8) | 7.1 (6 -8.2) | 0.29 |
| LAV (ml) | 59.9 (52.3 -67.5) | 54.5 (47.2 -61.9) | 0.17 | 65 (58.7 -71.3) | 58.5 (51.8 -65.2) | **<0.05** | 66.3 (60.3 -72.3) | 59.4 (52.8 -66) | **0.02** |
| LAV index (ml/BSA) | 26.3 (22.8 -29.7) | 25.7 (22.4 -29.1) | 0.78 | 28.3 (25.4 -31.3) | 28 (24.8 -31.1) | 0.85 | 29.2 (26.2 -32.2) | 28.4 (25.1 -31.6) | 0.58 |
| **Strain** |  | | | | | | | | |
| GLS (%) | -15.1 (-19-(-13.1)) | -16.6 (-19.5-(-13.6)) | 0.58 | -14.4 (-17.7- (-11.2)) | -17.3 (-20.3-(-14.4)) | 0.08 | -14.4 (-17.7-(-11.1)) | -17.6 (-20.8- (-14.3)) | 0.06 |
| GCS (%) | -23.1 (-27.7-(-18.5)) | -22.88 (-27.4-(-18.)) | 0.59 | -22.33 (-26.9-(-17.7)) | -21.9 (-26.6-(-19.2)) | 0.76 | -19.7 (-23.8- (-15.7)) | -23.3 (-28.0-(-20.4)) | **0.03** |

Data are given as estimated marginal means (95% CI) for each trimester and group with *P* values of group comparison. *BSA*, body surface area. **Haemodynamic:** *CO*, cardiac output; *CI* cardiac index; *DBP*, diastolic blood pressure; *HR*, heart rate; *MAP*, mean arterial pressure; *SV*, stroke volume; *SVI*, stroke volume index; *SBP*, systolic blood pressure; *PVR*, peripheral vascular resistance*.* **Geometry:** *IVS*, interventricular septum; *LA*, left atrium diameter; *LVEDD*, left ventricle end-diastolic diameter; *LVM*, left ventricular mass; *PW*, posterior wall thickness; *RWT*, relative wall thickness. **Systolic function:** *EF*, ejection fraction; *EDV*, end-diastolic volume; *ESV*, end-systolic volume; *LVEF*, left ventricle ejection fraction; *TDI s’*, tissue Doppler imaging s’ at the lateral tricuspid annulus. **Diastolic function:** *E/A ratio*, mitral inflow E-wave/A-wave filling; *E’ lateral*, tissue Doppler imaging E prime measured at lateral mitral annulus; *E’ medial*, tissue Doppler imaging E prime measured at medial mitral annulus; *E/E’ ratio*, E-wave mitral inflow/ mean E’ lateral and E’ medial; *LAV*, left atrial volume. **Strain:** *GCS,* global circumferential strain; *GLS*, global longitudinal strain

|  | **1st Trimester** | | | **2nd Trimester** | | | **3rd Trimester** | | |
| --- | --- | --- | --- | --- | --- | --- | --- | --- | --- |
|  | **No-surgery** | **Post-bariatric surgery** | **P** | **No-surgery** | **Post-bariatric surgery** | **P** | **No-surgery** | **Post-bariatric surgery** | **P** |
|  | **N=18** | **N=18** |  | **N=30** | **N=30** |  | **N=28** | **N=28** |  |
|  |  |  |  |  |  |  |  |  |  |
| SBP (mmHg) | 113.8 (107.3-130) | 101.5 (93.3-106.8) | **<0.001** | 112 (100.5-116.5) | 99 (91-109) | **<0.01** | 111 (103.5-121.5) | 103.5 (93-110) | **<0.01** |
| DBP (mmHg) | 62.8 (60-79) | 61 (59.3-66.3) | 0.15 | 65 (61-70.5) | 59 (55.5-62.3) | **<0.001** | 65 (60.5-71) | 62 (57.5-66.5) | **<0.05** |
| MAP (mmHg) | 81.4 (75.6-94.9) | 75 (69.5-79.5) | **<0.01** | 80.3 (75.8-86.7) | 74.3 (67.2-78.3) | **<0.001** | 80.1 (75.6-86.4) | 76.7 (70.3-80.7) | **0.02** |
| HR (bpm) | 83 (75.3-88.8) | 73 (65.5-75) | **<0.01** | 84 (74.5-93) | 75 (69-85.5) | **0.01** | 93.5 (83.8-98) | 80 (70-87) | **<0.001** |
| SV (ml) | 80.9 (72.5-92.2) | 72 (66.6-79.6) | **0.03** | 86.5 (72.2-104.6) | 79.6 (72-87.3) | **0.04** | 85.7 (77.3-99.7) | 80.5 (73-87.9) | 0.08 |
| CO (L/min) | 6.9 (6.3-7.4) | 5 (4.5-5.6) | **<0.001** | 7.5 (6.4-8.5) | 5.9 (5.3-6.9) | **<0.001** | 7.7 (6.3-9.2) | 6.2 (5.7-7.0) | **<0.01** |
| PVR (dynes/s per cm^5^) | 1002.9 (871.6-1158.9) | 1178.5 (990.6-1349.6) | **<0.05** | 874.7 (752-976.6) | 992.6 (856.7-1094.8) | 0.06 | 835.4 (675.6-986.4) | 971.5 (868.6-1083.9) | **<0.01** |
| SVI (mm/BSA) | 39.5 (30.8-43.8) | 36.6 (32.4-39.9) | 0.48 | 40 (36-48.3) | 39.5 (37.2-44.7) | 0.77 | 39.9 (35.4-45.6) | 40.5 (35-47.6) | 0.92 |
| CI (mm/BSA) | 3.2 (2.9-3.5) | 2.5 (2.3-2.9) | **<0.01** | 3.4 (2.9-3.8) | 3 (2.7-3.4) | **0.01** | 3.6 (3-4.3) | 3.2 (2.8-3.6) | **0.01** |

**Table S2** Hemodynamic parameters in no-surgery and postsurgery groups at each trimester

Data are given as median (interquartile range). *BSA*, body surface area; *CO*, cardiac output; *CI* cardiac index; *DBP*, diastolic blood pressure; *HR*, heart rate; *MAP*, mean arterial pressure; *PVR*, total peripheral resistance; *SV*, stroke volume; *SVI*, stroke volume index; *SBP*, systolic blood pressure
